# CHRONIC CONDITION PATTERNS IN THE U.S. POPULATION AND THEIR RELATIONSHIP WITH MORTALITY

**DOI:** 10.1093/geroni/igz038.1161

**Published:** 2019-11-08

**Authors:** D Diane Zheng, Sharon L Christ, Byron Lam, Kathryn McCollister, Daniel J Feaster, David J Lee

**Affiliations:** 1 University of Miami, Miami, Florida, United States; 2 Purdue University, West Lafayette, Indiana, United States; 3 Bascom Palmer Eye Institute, Miami, Florida, United States

## Abstract

Using data from 372,933 participants age >18 years from National Health Interview Survey 2002-2014, we employed latent class analysis to develop latent classes or subgroups of participants based on their combination of 13 self-reported chronic conditions. Mortality linkage with National Death Index was performed through December 31st, 2015. Survival analyses were conducted to assess how the derived latent class membership predicted all-cause mortality and cause specific mortalities. Five latent groups were identified with 70.5% of the participants belonging to the “healthy group”. The other four groups represented various degrees and patterns of multi-morbidity and were labeled accordingly: “hypertensive group” 20%, “respiratory condition group” 3.9%, “heart condition group” 3.7%, and “severely impaired group” 1.9%. 32,609 deaths were identified with average follow-up time of 6.93 years. After controlling for survey design, age, gender, race, Hispanic origin, education, income, health insurance, BMI, smoking and alcohol drinking status, compared to the healthy group, participants in all four latent disease groups had elevated mortality risk: hypertensive group Hazard Ratio(HR) 1.57 (95% confidence interval [1.49, 1.65]); respiratory condition group (2.08 [1.93, 2.24]); heart disease group (2.27 [2.13, 2.42]); and severely impaired group (3.84 [3.55, 4.16]; all p-values <0.01). Patterns of the chronic condition classes were also strongly associated with the primary underline cause of death. Four multi-morbidity groups, comprising 29.5% of the US population were at significantly elevated risk of mortality. Assessing patterns of disease co-occurrence in the US population may be useful for identifying individuals in need of targeted interventions to reduce mortality risk.

